# (*E*)-Methyl *N*′-(3-methoxy­benzyl­idene)hydrazinecarboxyl­ate

**DOI:** 10.1107/S1600536809033194

**Published:** 2009-08-26

**Authors:** Lu-Ping Lv, Wen-Bo Yu, Chun-Yu Huang, Wei-Wei Li, Xian-Chao Hu

**Affiliations:** a Department of Chemical Engineering, Hangzhou Vocational and Technical College, Hangzhou 310018, People’s Republic of China; b The Children’s Hospital of Zhejiang University, School of Medicine, Hangzhou 310003, People’s Republic of China; c Research Center of Analysis and Measurement, Zhejiang University of Technology, Hangzhou 310014, People’s Republic of China

## Abstract

The title compound, C_10_H_12_N_2_O_3_, crystallizes with two independent mol­ecules in the asymmetric unit which differ in the orientation of the meth­oxy group. Each independent mol­ecule adopts a *trans* configuration with respect to the C=N bond. In the crystal structure, mol­ecules are linked into chains running along [001] by N—H⋯O and N—H⋯N hydrogen bonds. In addition, an inter­molecular C—H⋯π inter­action is observed.

## Related literature

For general background to the properties of benzaldehyde­hydrazone derivatives, see: Parashar *et al.* (1988 ▶); Hadjoudis *et al.* (1987 ▶); Borg *et al.* (1999 ▶); Kahwa *et al.* (1986 ▶); Santos *et al.* (2001 ▶). For a related structure, see: Shang *et al.* (2007 ▶).
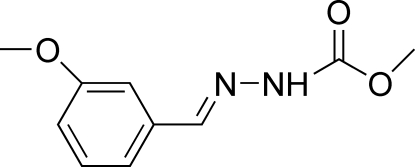


## Experimental

### 

#### Crystal data

C_10_H_12_N_2_O_3_
*M* *_r_* = 208.22
Monoclinic, 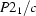

*a* = 18.207 (3) Å
*b* = 7.3677 (12) Å
*c* = 16.552 (3) Å
β = 104.243 (5)°
*V* = 2152.1 (6) Å^3^
*Z* = 8
Mo *K*α radiation
μ = 0.10 mm^−1^
*T* = 223 K
0.24 × 0.21 × 0.19 mm

#### Data collection

Bruker SMART CCD area-detector diffractometer
Absorption correction: multi-scan (*SADABS*; Bruker, 2002 ▶) *T*_min_ = 0.977, *T*_max_ = 0.989
11364 measured reflections
3790 independent reflections
3033 reflections with *I* > 2σ(*I*)
*R*_int_ = 0.020

#### Refinement

*R*[*F* ^2^ > 2σ(*F* ^2^)] = 0.037
*wR*(*F* ^2^) = 0.109
*S* = 1.07
3790 reflections
276 parameters
H-atom parameters constrained
Δρ_max_ = 0.20 e Å^−3^
Δρ_min_ = −0.17 e Å^−3^

### 

Data collection: *SMART* (Bruker, 2002 ▶); cell refinement: *SAINT* (Bruker, 2002 ▶); data reduction: *SAINT*; program(s) used to solve structure: *SHELXS97* (Sheldrick, 2008 ▶); program(s) used to refine structure: *SHELXL97* (Sheldrick, 2008 ▶); molecular graphics: *SHELXTL* (Sheldrick, 2008 ▶); software used to prepare material for publication: *SHELXTL*.

## Supplementary Material

Crystal structure: contains datablocks I, global. DOI: 10.1107/S1600536809033194/ci2891sup1.cif

Structure factors: contains datablocks I. DOI: 10.1107/S1600536809033194/ci2891Isup2.hkl

Additional supplementary materials:  crystallographic information; 3D view; checkCIF report

## Figures and Tables

**Table 1:** Hydrogen-bond geometry (Å, °)

*D*—H⋯*A*	*D*—H	H⋯*A*	*D*⋯*A*	*D*—H⋯*A*
N2—H2⋯O5	0.86	2.07	2.9188 (18)	172
N4—H4⋯O2^i^	0.86	2.25	2.8365 (17)	126
N4—H4⋯N1^i^	0.86	2.55	3.3883 (19)	164
C11—H11*B*⋯*Cg*1^ii^	0.96	2.91	3.776 (3)	150

Symmetry codes: (i) 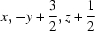; (ii) 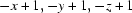. *Cg*1 is the centroid of the C12–C17 ring.

**Fig. 1.**
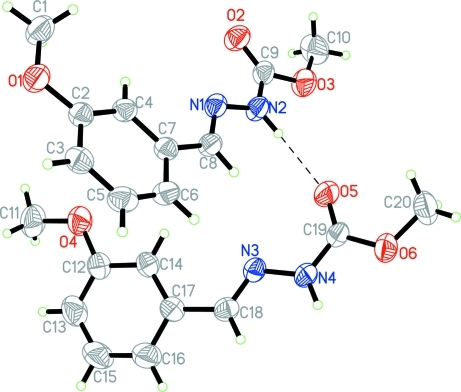
The asymmetric unit of the title compound. Displacement ellipsoids are drawn at the 40% probability level. The dashed line indicates a hydrogen bond.

**Fig. 2.**
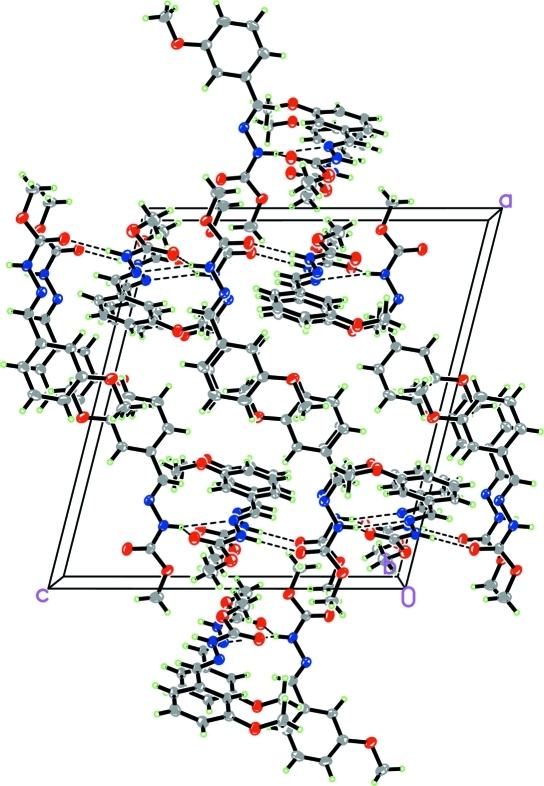
Crystal packing of the title compound. Hydrogen bonds are shown as dashed lines.

## supplementary crystallographic information

### Comment

Benzaldehydehydrazone derivatives have attracted much attention due to their pharmacological activity (Parashar *et al.*, 1988) and their photochromic properties (Hadjoudis *et al.*, 1987). They are important intermediates of 1,3,4-oxadiazoles, which have been reported to be versatile compounds with many interesting properties (Borg *et al.*, 1999). Metal complexes based on Schiff bases have received considerable attention because they can be utilized as model compounds of active centres in various proteins and enzymes (Kahwa *et al.*, 1986; Santos *et al.*, 2001). We report here the crystal structure of the title compound (Fig. 1).

The title compound contains two independent, but almost identical molecules in the asymmetric unit. Each independent molecule adopts a *trans* configuration with respect to the C═N bond. The N1/N2/O2/O3/C8/C9/C10 and N3/N4/O5/O6/C18/C19/C20 planes form dihedral angles of 4.51 (6)° and 13.80 (6)°, respectively, with the C2—C7 and C12—C17 planes. The dihedral angle between the two independent benzene rings is 59.18 (6)°. Bond lengths and angles are comparable to those observed for methyl*N*'-[(*E*)-4-methoxybenzylidene]hydrazinecarboxylate (Shang *et al.*, 2007).

In the crystal structure, molecules are linked into chains running along the [001] by N—H···O and N—H···N hydrogen bonds. In addition, an intermolecular C—H···π interaction is observed (Table 1).

### Experimental

3-Methoxybenzaldehyde (1.36 g, 0.01 mol) and methyl hydrazinecarboxylate (0.90 g, 0.01 mol) were dissolved in stirred methanol (20 ml) and left for 2.5 h at room temperature. The resulting solid was filtered off and recrystallized from ethanol to give the title compound in 95% yield. Single crystals suitable for X-ray analysis were obtained by slow evaporation of an ethanol solution at room temperature (m.p. 436–438 K).

### Refinement

H atoms were positioned geometrically (N-H = 0.86 Å and C-H = 0.93 or 0.96 Å) and refined using a riding model, with *U*_iso_(H) = 1.2*U*_eq_(C,N) and 1.5*U*_eq_(C_methyl_). A rotating group model was used for the methyl groups.

### Crystal data

**Table:**

C_10_H_12_N_2_O_3_	*F*(000) = 880
*M**_r_* = 208.22	*D*_x_ = 1.285 Mg m^−^^3^
Monoclinic, *P*2_1_/*c*	Mo *K*α radiation, λ = 0.71073 Å
Hall symbol: -P 2ybc	Cell parameters from 3790 reflections
*a* = 18.207 (3) Å	θ = 2.3–25.0°
*b* = 7.3677 (12) Å	µ = 0.10 mm^−^^1^
*c* = 16.552 (3) Å	*T* = 223 K
β = 104.243 (5)°	Block, colourless
*V* = 2152.1 (6) Å^3^	0.24 × 0.21 × 0.19 mm
*Z* = 8	

### Data collection

**Table:**

Bruker SMART CCD area-detector diffractometer	3790 independent reflections
Radiation source: fine-focus sealed tube	3033 reflections with *I* > 2σ(*I*)
graphite	*R*_int_ = 0.020
φ and ω scans	θ_max_ = 25.0°, θ_min_ = 2.3°
Absorption correction: multi-scan (*SADABS*; Bruker, 2002)	*h* = −21→18
*T*_min_ = 0.977, *T*_max_ = 0.989	*k* = −8→8
11364 measured reflections	*l* = −19→19

### Refinement

**Table:**

Refinement on *F*^2^	Secondary atom site location: difference Fourier map
Least-squares matrix: full	Hydrogen site location: inferred from neighbouring sites
*R*[*F*^2^ > 2σ(*F*^2^)] = 0.037	H-atom parameters constrained
*wR*(*F*^2^) = 0.109	*w* = 1/[σ^2^(*F*_o_^2^) + (0.054*P*)^2^ + 0.3311*P*] where *P* = (*F*_o_^2^ + 2*F*_c_^2^)/3
*S* = 1.07	(Δ/σ)_max_ = 0.004
3790 reflections	Δρ_max_ = 0.20 e Å^−^^3^
276 parameters	Δρ_min_ = −0.17 e Å^−^^3^
0 restraints	Extinction correction: *SHELXL97* (Sheldrick, 2008), Fc^*^=kFc[1+0.001xFc^2^λ^3^/sin(2θ)]^-1/4^
Primary atom site location: structure-invariant direct methods	Extinction coefficient: 0.0115 (12)

### Special details

**Table:**

Geometry. All esds (except the esd in the dihedral angle between two l.s. planes) are estimated using the full covariance matrix. The cell esds are taken into account individually in the estimation of esds in distances, angles and torsion angles; correlations between esds in cell parameters are only used when they are defined by crystal symmetry. An approximate (isotropic) treatment of cell esds is used for estimating esds involving l.s. planes.
Refinement. Refinement of F^2^ against ALL reflections. The weighted R-factor wR and goodness of fit S are based on F^2^, conventional R-factors R are based on F, with F set to zero for negative F^2^. The threshold expression of F^2^ > 2sigma(F^2^) is used only for calculating R-factors(gt) etc. and is not relevant to the choice of reflections for refinement. R-factors based on F^2^ are statistically about twice as large as those based on F, and R- factors based on ALL data will be even larger.

### Fractional atomic coordinates and isotropic or equivalent isotropic displacement parameters (Å^2^)

**Table:**

	*x*	*y*	*z*	*U*_iso_*/*U*_eq_	
O1	0.68188 (7)	1.33813 (15)	−0.17091 (7)	0.0626 (3)	
O2	0.86739 (7)	0.49630 (16)	−0.12622 (7)	0.0661 (3)	
O3	0.92117 (7)	0.34574 (16)	−0.00794 (8)	0.0717 (4)	
O4	0.55036 (7)	0.73143 (19)	−0.03581 (8)	0.0788 (4)	
O5	0.90715 (7)	0.5763 (2)	0.18325 (7)	0.0792 (4)	
O6	0.96333 (6)	0.64573 (18)	0.31586 (7)	0.0690 (4)	
N1	0.82614 (7)	0.75999 (16)	−0.02931 (7)	0.0491 (3)	
N2	0.86458 (7)	0.60387 (17)	0.00177 (8)	0.0545 (3)	
H2	0.8769	0.5836	0.0546	0.065*	
N3	0.77335 (7)	0.69523 (16)	0.21361 (7)	0.0492 (3)	
N4	0.84157 (7)	0.68885 (19)	0.27209 (8)	0.0574 (4)	
H4	0.8442	0.7210	0.3227	0.069*	
C1	0.69744 (12)	1.2445 (3)	−0.23962 (10)	0.0724 (5)	
H1A	0.6782	1.3132	−0.2896	0.109*	
H1B	0.6735	1.1276	−0.2449	0.109*	
H1C	0.7512	1.2294	−0.2309	0.109*	
C2	0.70930 (8)	1.2656 (2)	−0.09344 (9)	0.0482 (4)	
C3	0.70053 (9)	1.3743 (2)	−0.02757 (10)	0.0559 (4)	
H3	0.6774	1.4873	−0.0382	0.067*	
C4	0.74387 (8)	1.0972 (2)	−0.07774 (9)	0.0478 (4)	
H4A	0.7500	1.0249	−0.1217	0.057*	
C5	0.72602 (10)	1.3148 (2)	0.05299 (10)	0.0612 (4)	
H5	0.7204	1.3879	0.0969	0.073*	
C6	0.76013 (9)	1.1460 (2)	0.06930 (10)	0.0567 (4)	
H6	0.7768	1.1058	0.1240	0.068*	
C7	0.76944 (8)	1.0368 (2)	0.00429 (9)	0.0475 (4)	
C8	0.80804 (9)	0.8620 (2)	0.02498 (9)	0.0510 (4)	
H8	0.8199	0.8239	0.0802	0.061*	
C9	0.88263 (9)	0.4835 (2)	−0.05143 (10)	0.0522 (4)	
C10	0.94571 (13)	0.2072 (3)	−0.05631 (15)	0.0888 (6)	
H10A	0.9814	0.1291	−0.0201	0.133*	
H10B	0.9694	0.2625	−0.0960	0.133*	
H10C	0.9028	0.1373	−0.0853	0.133*	
C11	0.47641 (11)	0.7249 (3)	−0.09038 (13)	0.0860 (6)	
H11A	0.4804	0.7021	−0.1463	0.129*	
H11B	0.4513	0.8388	−0.0885	0.129*	
H11C	0.4478	0.6293	−0.0731	0.129*	
C12	0.55619 (9)	0.7559 (2)	0.04734 (10)	0.0567 (4)	
C13	0.49554 (11)	0.7833 (3)	0.08170 (14)	0.0777 (6)	
H13	0.4464	0.7862	0.0480	0.093*	
C14	0.62919 (9)	0.7513 (2)	0.09829 (10)	0.0518 (4)	
H14	0.6700	0.7337	0.0747	0.062*	
C15	0.50852 (12)	0.8064 (4)	0.16661 (15)	0.0962 (7)	
H15	0.4676	0.8253	0.1899	0.115*	
C16	0.58066 (11)	0.8024 (3)	0.21795 (13)	0.0812 (6)	
H16	0.5882	0.8194	0.2751	0.097*	
C17	0.64204 (9)	0.7725 (2)	0.18355 (10)	0.0531 (4)	
C18	0.71842 (9)	0.7617 (2)	0.23885 (10)	0.0528 (4)	
H18	0.7269	0.8041	0.2933	0.063*	
C19	0.90395 (9)	0.6325 (2)	0.25016 (9)	0.0504 (4)	
C20	1.03667 (10)	0.6043 (3)	0.30340 (14)	0.0839 (6)	
H20A	1.0750	0.6610	0.3463	0.126*	
H20B	1.0401	0.6489	0.2500	0.126*	
H20C	1.0441	0.4752	0.3056	0.126*	

### Atomic displacement parameters (Å^2^)

**Table:**

	*U*^11^	*U*^22^	*U*^33^	*U*^12^	*U*^13^	*U*^23^
O1	0.0750 (8)	0.0619 (7)	0.0499 (6)	0.0148 (6)	0.0135 (5)	0.0052 (5)
O2	0.0832 (8)	0.0608 (7)	0.0546 (7)	0.0141 (6)	0.0177 (6)	0.0080 (5)
O3	0.0808 (9)	0.0605 (7)	0.0716 (8)	0.0215 (6)	0.0150 (6)	0.0188 (6)
O4	0.0582 (8)	0.1052 (10)	0.0640 (8)	0.0062 (7)	−0.0020 (6)	−0.0096 (7)
O5	0.0762 (9)	0.1133 (11)	0.0448 (7)	0.0302 (8)	0.0089 (6)	−0.0079 (7)
O6	0.0538 (7)	0.0895 (9)	0.0555 (7)	0.0082 (6)	−0.0021 (5)	−0.0090 (6)
N1	0.0505 (8)	0.0482 (7)	0.0468 (7)	0.0021 (6)	0.0085 (6)	0.0069 (5)
N2	0.0622 (8)	0.0538 (7)	0.0444 (7)	0.0074 (6)	0.0071 (6)	0.0094 (6)
N3	0.0509 (8)	0.0497 (7)	0.0434 (7)	−0.0003 (6)	0.0045 (6)	−0.0027 (5)
N4	0.0532 (8)	0.0758 (9)	0.0387 (7)	0.0044 (7)	0.0026 (6)	−0.0129 (6)
C1	0.0914 (14)	0.0770 (12)	0.0465 (9)	0.0169 (10)	0.0127 (9)	0.0039 (8)
C2	0.0458 (9)	0.0518 (8)	0.0477 (8)	−0.0018 (7)	0.0126 (6)	0.0009 (6)
C3	0.0568 (10)	0.0507 (8)	0.0629 (10)	0.0035 (7)	0.0201 (8)	−0.0039 (7)
C4	0.0493 (9)	0.0511 (8)	0.0437 (8)	−0.0004 (7)	0.0129 (6)	−0.0042 (6)
C5	0.0685 (11)	0.0645 (10)	0.0544 (10)	0.0014 (9)	0.0226 (8)	−0.0122 (8)
C6	0.0589 (10)	0.0689 (10)	0.0438 (8)	−0.0003 (8)	0.0157 (7)	−0.0013 (7)
C7	0.0432 (8)	0.0536 (8)	0.0468 (8)	−0.0038 (7)	0.0132 (6)	0.0006 (6)
C8	0.0519 (9)	0.0573 (9)	0.0431 (8)	−0.0014 (7)	0.0104 (7)	0.0057 (7)
C9	0.0507 (9)	0.0493 (8)	0.0546 (9)	0.0015 (7)	0.0091 (7)	0.0112 (7)
C10	0.0927 (15)	0.0646 (11)	0.1145 (17)	0.0286 (11)	0.0360 (13)	0.0127 (11)
C11	0.0681 (13)	0.0987 (15)	0.0760 (13)	−0.0005 (11)	−0.0113 (10)	−0.0085 (11)
C12	0.0513 (10)	0.0520 (9)	0.0634 (11)	−0.0023 (7)	0.0077 (8)	−0.0035 (7)
C13	0.0454 (10)	0.0951 (14)	0.0882 (15)	−0.0051 (10)	0.0079 (9)	−0.0067 (11)
C14	0.0457 (9)	0.0492 (8)	0.0596 (9)	−0.0009 (7)	0.0112 (7)	−0.0031 (7)
C15	0.0515 (12)	0.152 (2)	0.0911 (16)	−0.0039 (13)	0.0284 (11)	−0.0137 (15)
C16	0.0600 (12)	0.1185 (17)	0.0685 (12)	−0.0066 (11)	0.0224 (10)	−0.0107 (11)
C17	0.0496 (9)	0.0503 (8)	0.0601 (10)	−0.0052 (7)	0.0149 (7)	−0.0030 (7)
C18	0.0571 (10)	0.0539 (9)	0.0467 (8)	−0.0048 (7)	0.0116 (7)	−0.0053 (7)
C19	0.0565 (10)	0.0491 (8)	0.0414 (8)	0.0052 (7)	0.0044 (7)	0.0021 (6)
C20	0.0541 (12)	0.0970 (15)	0.0941 (15)	0.0160 (10)	0.0058 (10)	0.0073 (12)

### Geometric parameters (Å, °)

**Table:**

O1—C2	1.3654 (18)	C5—C6	1.387 (2)
O1—C1	1.417 (2)	C5—H5	0.93
O2—C9	1.2039 (18)	C6—C7	1.387 (2)
O3—C9	1.3387 (18)	C6—H6	0.93
O3—C10	1.435 (2)	C7—C8	1.467 (2)
O4—C12	1.366 (2)	C8—H8	0.93
O4—C11	1.426 (2)	C10—H10A	0.96
O5—C19	1.1971 (18)	C10—H10B	0.96
O6—C19	1.3351 (18)	C10—H10C	0.96
O6—C20	1.433 (2)	C11—H11A	0.96
N1—C8	1.2757 (19)	C11—H11B	0.96
N1—N2	1.3785 (17)	C11—H11C	0.96
N2—C9	1.346 (2)	C12—C13	1.376 (3)
N2—H2	0.86	C12—C14	1.388 (2)
N3—C18	1.273 (2)	C13—C15	1.377 (3)
N3—N4	1.3749 (17)	C13—H13	0.93
N4—C19	1.341 (2)	C14—C17	1.381 (2)
N4—H4	0.86	C14—H14	0.93
C1—H1A	0.96	C15—C16	1.378 (3)
C1—H1B	0.96	C15—H15	0.93
C1—H1C	0.96	C16—C17	1.391 (2)
C2—C4	1.386 (2)	C16—H16	0.93
C2—C3	1.393 (2)	C17—C18	1.467 (2)
C3—C5	1.372 (2)	C18—H18	0.93
C3—H3	0.93	C20—H20A	0.96
C4—C7	1.396 (2)	C20—H20B	0.96
C4—H4A	0.93	C20—H20C	0.96
			
C2—O1—C1	117.64 (12)	O3—C10—H10A	109.5
C9—O3—C10	115.68 (14)	O3—C10—H10B	109.5
C12—O4—C11	118.03 (15)	H10A—C10—H10B	109.5
C19—O6—C20	117.62 (14)	O3—C10—H10C	109.5
C8—N1—N2	115.11 (12)	H10A—C10—H10C	109.5
C9—N2—N1	119.26 (12)	H10B—C10—H10C	109.5
C9—N2—H2	120.4	O4—C11—H11A	109.5
N1—N2—H2	120.4	O4—C11—H11B	109.5
C18—N3—N4	115.39 (13)	H11A—C11—H11B	109.5
C19—N4—N3	119.91 (12)	O4—C11—H11C	109.5
C19—N4—H4	120.0	H11A—C11—H11C	109.5
N3—N4—H4	120.0	H11B—C11—H11C	109.5
O1—C1—H1A	109.5	O4—C12—C13	124.38 (16)
O1—C1—H1B	109.5	O4—C12—C14	115.73 (15)
H1A—C1—H1B	109.5	C13—C12—C14	119.90 (16)
O1—C1—H1C	109.5	C12—C13—C15	119.10 (17)
H1A—C1—H1C	109.5	C12—C13—H13	120.4
H1B—C1—H1C	109.5	C15—C13—H13	120.4
O1—C2—C4	124.80 (13)	C17—C14—C12	120.93 (16)
O1—C2—C3	115.07 (13)	C17—C14—H14	119.5
C4—C2—C3	120.13 (14)	C12—C14—H14	119.5
C5—C3—C2	120.02 (15)	C13—C15—C16	121.65 (19)
C5—C3—H3	120.0	C13—C15—H15	119.2
C2—C3—H3	120.0	C16—C15—H15	119.2
C2—C4—C7	119.68 (14)	C15—C16—C17	119.41 (19)
C2—C4—H4A	120.2	C15—C16—H16	120.3
C7—C4—H4A	120.2	C17—C16—H16	120.3
C3—C5—C6	120.27 (15)	C14—C17—C16	119.00 (16)
C3—C5—H5	119.9	C14—C17—C18	121.84 (15)
C6—C5—H5	119.9	C16—C17—C18	119.15 (16)
C7—C6—C5	120.23 (15)	N3—C18—C17	121.13 (14)
C7—C6—H6	119.9	N3—C18—H18	119.4
C5—C6—H6	119.9	C17—C18—H18	119.4
C6—C7—C4	119.66 (14)	O5—C19—O6	124.39 (15)
C6—C7—C8	118.10 (13)	O5—C19—N4	126.52 (14)
C4—C7—C8	122.22 (14)	O6—C19—N4	109.08 (13)
N1—C8—C7	123.03 (13)	O6—C20—H20A	109.5
N1—C8—H8	118.5	O6—C20—H20B	109.5
C7—C8—H8	118.5	H20A—C20—H20B	109.5
O2—C9—O3	124.75 (15)	O6—C20—H20C	109.5
O2—C9—N2	126.11 (14)	H20A—C20—H20C	109.5
O3—C9—N2	109.14 (13)	H20B—C20—H20C	109.5
			
C8—N1—N2—C9	176.68 (14)	N1—N2—C9—O3	178.40 (12)
C18—N3—N4—C19	−175.58 (14)	C11—O4—C12—C13	−3.2 (3)
C1—O1—C2—C4	−7.6 (2)	C11—O4—C12—C14	176.59 (15)
C1—O1—C2—C3	172.35 (15)	O4—C12—C13—C15	180.0 (2)
O1—C2—C3—C5	179.99 (15)	C14—C12—C13—C15	0.2 (3)
C4—C2—C3—C5	−0.1 (2)	O4—C12—C14—C17	−179.29 (14)
O1—C2—C4—C7	−179.72 (14)	C13—C12—C14—C17	0.5 (2)
C3—C2—C4—C7	0.4 (2)	C12—C13—C15—C16	−0.2 (4)
C2—C3—C5—C6	−0.4 (3)	C13—C15—C16—C17	−0.6 (4)
C3—C5—C6—C7	0.7 (3)	C12—C14—C17—C16	−1.3 (2)
C5—C6—C7—C4	−0.4 (2)	C12—C14—C17—C18	177.79 (14)
C5—C6—C7—C8	178.06 (15)	C15—C16—C17—C14	1.3 (3)
C2—C4—C7—C6	−0.1 (2)	C15—C16—C17—C18	−177.8 (2)
C2—C4—C7—C8	−178.51 (13)	N4—N3—C18—C17	−178.49 (13)
N2—N1—C8—C7	177.72 (13)	C14—C17—C18—N3	−15.0 (2)
C6—C7—C8—N1	−172.84 (15)	C16—C17—C18—N3	164.04 (17)
C4—C7—C8—N1	5.6 (2)	C20—O6—C19—O5	5.5 (2)
C10—O3—C9—O2	0.7 (2)	C20—O6—C19—N4	−175.48 (15)
C10—O3—C9—N2	−178.90 (15)	N3—N4—C19—O5	−3.8 (3)
N1—N2—C9—O2	−1.2 (2)	N3—N4—C19—O6	177.20 (13)

### Hydrogen-bond geometry (Å, °)

**Table:**

*D*—H···*A*	*D*—H	H···*A*	*D*···*A*	*D*—H···*A*
N2—H2···O5	0.86	2.07	2.9188 (18)	172
N4—H4···O2^i^	0.86	2.25	2.8365 (17)	126
N4—H4···N1^i^	0.86	2.55	3.3883 (19)	164
C11—H11B···Cg1^ii^	0.96	2.91	3.776 (3)	150

Symmetry codes: (i) *x*, −*y*+3/2, *z*+1/2; (ii) −*x*+1, −*y*+1, −*z*+1.
